# Deep learning-based early prediction of life-threatening ventricular arrhythmias using long-term Holter ECG signals

**DOI:** 10.3389/fcvm.2026.1714589

**Published:** 2026-02-06

**Authors:** Yifan Wu, Yu Chen, Bin Zhang, Xusong Chen, Chang Peng, Jin Jiang, Jianming Chen, Chunyan Jian, Guozhi Wu

**Affiliations:** 1 Department of Cardiology, Central People’s Hospital of Zhanjiang, Zhanjiang, China; 2 Shenzhen Dawei Medical Technology Development Co., Ltd., Shenzhen, China; 3 Department of Cardiovascular Diagnostics, Central People’s Hospital of Zhanjiang, Zhanjiang, China; 4 Department of the First Ward, Intensive Care Medicine Unit, Central People’s Hospital of Zhanjiang, Zhanjiang, China

**Keywords:** arrhythmia detection, graph neural networks, Holter ECG signals, transformer encoder, ventricular arrhythmias

## Abstract

**Introduction:**

Ventricular arrhythmias (VAs) are among the primary reasons for sudden cardiac death, and their early detection requires a key factor to reduce patient mortality. Conventional tools used to identify arrhythmias (manual and rule-based) are not only time-consuming but also have become dependent on an expert to interpret; thus, their utility is constrained in terms of their scalability and applicability in viewing arrhythmias in real-time. The increasing rate of cardiovascular diseases (CVD) and the desire to have an efficient and real-time environment are evidenced in the weaknesses of current systems.

**Methods:**

The current research introduces a new deep learning framework on the basis of graph neural networks (GNNs) and a transformer to improve the detection of arrhythmia with the Holter ECG signal. The data are collected via Holter ECGs and the Sudden Cardiac Death Holter Database (SDDB) as a basis to start the workflow. It involves preprocessing of data, such as elimination of noise, normalization of the signals, and segmentation of the data to pertinent ECG segments. Features are then extracted by a combination of time domain, frequency domain, and non-linear techniques, followed by classification using the hybrid GNN + transformer model to incorporate both the spatial and the temporal dependencies. In comparison to classical methods of the rule -based approaches, machine learning algorithms, such as the hidden Markov models (CNN-Bi-LSTM) and recurrent neural networks (Bi-LSTM), the hybrid model of GNN + transformer automatically determines arrhythmias, including spatial and temporal dependencies, to enhance the classification accuracy by a significant margin.

**Results:**

Model training and testing were performed on MIT-BIH and SDDB, and the accuracy, precision, recall, and F1-score were 98.27%, 98.08%, 98.27%, and 97.76%, respectively. This evidence proves the framework to be powerful in practical arrhythmia identification, providing a trustworthy way of monitoring the heart.

**Discussion:**

The hybrid model is efficient compared with the traditional models and offers an extensible solution to wearable healthcare systems that would have the quality of detecting arrhythmia in real-time with a high degree of accuracy.

## 1 Introduction

Ventricular arrhythmias (VAs) are a significant cause of sudden cardiac death (SCD) and an enormous burden on health systems due to their prevalence in worldwide settings (1). Such dangerous cardiac arrhythmias are life-threatening, and it is essential to identify them as early as possible to avoid death and optimize the clinical outcome of patients (2). The modern diagnostic technique commonly used, including the technology of monitoring based on ECG, is widely ineffective and restricted by requiring professional explanation (3). In addition, the existing systems are mostly reactive, which means they identify the arrhythmias after they happen and, thus, they fail to intervene at the right time (4). This is especially significant in at-risk populations where early intervention is essential to survival (5). However, with the current trends of machine learning (ML) and deep learning (DL), a new scope exists to enhance early detection by using the long-term Holter ECG signal processed by automated and real-time monitoring (6).

Moreover, the rising rate of cardiovascular diseases (CVD), especially in the populated areas with an ageing population, has further contributed to increasing the pressure of having effective arrhythmia detection systems (7). In some of these countries, such as China, where the burden of cardiovascular health is increasing, it is imperative to develop systems capable of analyzing data related to ECGs captured by wearable devices in short amounts of time (8). To treat ventricular arrhythmias, implantable cardioverter defibrillators (ICDs) were created along with subcutaneous ICD (S-ICD), as they manage to treat arrhythmias after they have already happened, which is too late in many cases (9). The suggested framework, composed of deep learning algorithms, is supposed to fill this gap as it will give early diagnosis and classification of arrhythmias, which in turn will allow early intervention and improved clinical outcomes (10).

Algorithms developed so far to detect the arrhythmia are mostly based on ML, including support vector machines (SVM), random forest (RF), and k-nearest neighbors (k-NN) that have been used to classify ECG signal (10, 11). Such methods usually take a set of hand-built features extracted from raw ECG data and classify (12). Although the methods have proved to be effective to some degree, they are restricted by the fact that they are dependent on handcrafted features that do not necessarily demonstrate the complete complexity of the ECG signals (13). Such methods may also suffer class imbalance problems, in which life-threatening arrhythmia is highly underrepresented compared with normal heart rhythms (14). Consequently, they might not make up valid forecasts on rare events such as ventricular fibrillation and ventricular tachycardia (15).

The other typical solution is employing convolutional neural networks (CNNs) and long short-term memory (LSTM) networks, which are better at capturing space-time characteristics of ECG signals (16). CNNs are adept at feature extraction that is automated on the data, and LSTMs model sequential dependencies across time (17). Yet these models tend to be overfitted especially when trained over small data and may be susceptible to generalizing to unseen data or other patient groups (18). Moreover, these solutions can be coupled with the multi-lead ECG data that may make these approaches more difficult as the models have to learn complex relationships between the leads, not necessarily easily represented (19).

The framework, proposed, can mitigate the weaknesses of current systems since it integrates the advantages of graph neural networks (GNNs) and transformer architectures. The inter-lead dependencies between several ECG leads are captured with GNNs by handling the multidimensionality of multi-lead ECG signals. Transformer encoder layers, which are notable to model long-range dependencies, enable the framework to concentrate on significant components of the ECG signal that could refer to arrhythmic events. Incorporation of the two versions enables the system to learn the spatial and temporal features, which strengthens the generalizability and detects arrhythmia of high standards through the model. The application of the self-attention mechanism to the transformer layer also indicates that the problem under consideration will prioritize the most relevant segments of the ECG signal, and then the accuracy of detection will be improved with regard to life-threatening arrhythmias.

The uniqueness of the research is the fact that it is the first study that integrates GNNs and transformers into a single network to deal with the problem of arrhythmia detection in Holter ECG data. In addition to increasing the power of the model to deal with multi-lead ECG data, the composite model is also more predictive, adding to the strengths of both models. Moreover, the model is resistant to data imbalance and has the possibility of performing in real-time, which makes it applicable in continuous monitoring devices. This method represents an important step up in the detection of arrhythmias, offering a complete package toward the early diagnosis, prevention, and removal of the issue, particularly when it comes to the population that experiences the problem recurrently with high severity levels, since urgent treatment in such cases is paramount.

### 1.1 Objectives

Develop a DL-based framework for the early prediction of life-threatening VAs from long-term Holter ECG signals, aiming to improve real-time clinical decision-making and patient outcomes, particularly in high-risk populations.
Utilize the Sudden Cardiac Death Holter Database (SDDB) and other relevant Holter ECG datasets, ensuring comprehensive and region-specific data, such as from the Chinese population, to train the proposed model.
Implement GNNs to capture inter-lead dependencies between multiple ECG leads, addressing the spatial complexity of multi-lead ECG signals for arrhythmia detection.
Leverage transformer architectures with a self-attention mechanism to model long-range temporal dependencies and improve detection accuracy, focusing on crucial segments of the ECG signal indicative of arrhythmias.

The structure of the paper is as follows. Section 2 is a very informative review of existing work in the field of detecting arrhythmia by deep learning techniques, including the identification of different approaches and their limitations. Section 3 provides the description of GNNs and transformer architectures integration and how the combination of them allows the recognition of the spatial and temporal relationships of the ECG data so that the model can determine the arrhythmia of a patient. In Section 4, model performance is assessed through a number of metrics and compared with previously known methods, addressing the increase in detection accuracy and model robustness. Lastly, Section 5 summarizes the main discoveries in the study, highlights how the proposed framework can work significantly, and proposes future studies.

## 2 Related works

A literature survey entails a description of work conducted in a certain area and the methodologies applied. It will assist in determining the level of knowledge, difficulties, and gaps that exist in the field. Its application through review of relevant research enables it to shape the creation of new models and methods to deal with these problems in a proper way.

Cai et al. (20) propose a lightweight and interpretable model to identify life-threatening VAs on short-term single-lead ECG signals, which overcomes the drawbacks of the older methods in terms of being computationally expensive and inapplicability in real-time. The method achieves analysis using a fixed-frequency-range empirical wavelet transform and uses multiscale visibility graphs and recurrence networks to process the signals and then use XGBoost classifier. It reaches high sensitivity (99.02%), specificity (98.44%), and accuracy (98.73%) at 10 s segment length and shows good values on shorter 2 s segments as well. The approach also achieves high performance compared with current feature-based and deep learning models and is both interpretable and implementable in real-time mobile settings, day-to-day care in wearable healthcare. Vásquez-Iturralde et al. (21) offer PVCNet, a deep learning model that classifies premature ventricular contractions (PVCs) among patients with single-lead electrocardiograph records despite a data imbalance and mislabeled segments. The cutting-edge method applies sequence heartbeats as labels to segments to enhance quality information and labeling and retrains the loss function to focus on the detection of specific heartbeats. PVCNet presents high implementations on the testing database, which shows its ability to implement long-term monitoring of ECG. Such findings emphasize its possible use in detecting PVC in clinical practice as a useful instrument for observing the changes.

Wang et al. (22) propose an automatic end-to-end DL approach based on a DenseNet network model to diagnose cardiac arrhythmias on an imbalanced ECG dataset based on the need to increase diagnosis effectiveness and decrease the number of tasks in the hands of doctors. The model is trained with the MIT-BIH and INCART datasets and is trained to classify ECG signals in four classes. In experimental results, the model presents better results compared with existing ones. This effectiveness is also confirmed by transfer learning, which proves the feasibility of its use in practice in identifying arrhythmia even in the presence of a class imbalance. Mohanty et al. (23) offer a new approach to ventricular arrhythmias, ventricular tachycardia (VT) and ventricular fibrillation (VF), heart-threatening ventricular arrhythmias, and orthodox ventricular arrhythmias. C4.5 classifier is more effective than SVM because it has high sensitivity, specificity, and accuracy to act as a clinical decision support in detecting arrhythmia.

Kim et al. (24) present a new hybrid CNN–transformer architecture to distinguish arrhythmia using ECG signals using the Stockwell transform as an effective feature extraction tool that does not need to identify the R-peak. Compared with traditional CNN-based models, the model enhances the efficiency and accuracy of recognizing arrhythmia. It shows good results on different ECG datasets such as Icentia11k and MIT-BIH, with high rates of classification accuracy in different types of arrhythmias. The suggested methodology can augment the diagnosis of arrhythmia using ECG, and it is able to fit applications in real-time monitoring systems. Chen et al. (25) proposed a PAFNet model that employs a deep learning framework and a sliding-window process applied to crude RR intervals of ECG segments to forecast the occurrence of paroxysmal atrial fibrillation (PAF), corroboration in real-time. The model is employed by considering a convolutional neural network (CNN) with different sizes of RR intervals, and the model exhibits the best results with 100 intervals. It shows good prediction accuracy, sensitivity, and specificity and proves effective in the identification of the onset of PAF. The model analyzes the samples fairly quickly, and with the anticipation of at least 45 min, it can indicate PAF, which is an important point concerning real-time clinical surveillance.

Randazzo et al. (26) propose a vision transformer (ViT) model to forecast that fatal arrhythmia will occur in patients with Brugada syndrome (BrS) by estimating the probability of an arrhythmic event based on the 12-lead ECG images, with the hope of supplementing risk stratification. To optimize the model, a set of 278 ECGs belonging to 210 patients with BrS who were diagnosed with the condition was used as a training set. The ViT model has a satisfactory performance since it has significant accuracy, sensitivity, and specificity on both balanced and unbalanced datasets. This methodology proves that deep learning has the potential to enhance arrhythmic risk determination and clinically facilitate BrS patients. Feng et al. (27) propose a new segmentation group of deep learning in detecting atrial fibrillation (AF) based on RR interval data, within which a one-dimensional UNet model is used. It had high sensitivity, specificity, and accuracy and showed consistent performance results when trained and tested using an in-house dataset of 200 24 h 12-lead Holter records. It overtakes other currently in use schemes of classification, which are based on datasets as it surpasses the results of the non-public and in-house datasets. The current approach demonstrates how it can be used in real clinical applications in the detection of AF, overcoming the shortcomings that have been experienced using previous methods.

Bashar et al. (28) introduce an automated approach toward the prediction of atrial fibrillation (AF) in sepsis patients in the intensive care unit (ICU) with an outcome that can allow an early prediction of AF by using ECG signal. The technique identifies characteristics of heart rate (HR) signals by the classical time, frequency domain, and non-linear domain approaches to feature extraction and novel tools. Setting the 2001 Computers in Cardiology as the training data, SVM and random forest were used to classify the data into the presence or absence of hyperkalemia and then tested with the status of data in MIMIC III, which yielded high values of sensitivity, specificity, and prediction. This algorithm was better than the current options and showed that the algorithm might be effective in predicting AF and intervene in an early time inside the ICUs. Akalın et al. (29) emphasize the application of ECG as a non-invasive device to identify the case of abnormal heart rhythm in a child. This model was then tested on an even more complicated data set consisting of six categories and proved to be relevant and sound against various and rich sets of data.

Muhammad et al. (30) introduce a two-stage multi-class algorithm of automatic CVD diagnostics based on ECG data through which, at the first stage, the ECG segmentation is realized with the help of a convolutional bidirectional LSTM with attention mechanism and the second stage of classification of ECG beats is performed with time-adaptive CNNs. The model showed high accuracy after being tested and trained on the MIT-BIH-PhysioNet databases, and this is in comparison with the traditional rule-based systems. Its performance in aiding clinical diagnosis proved to be very effective with the results showing that many improvements can be made through it over current algorithms.

### 2.1 Research gap

Detection of arrhythmia using ECG signals through current methods has a number of challenges. These methods (traditional approaches) tend to require intense interpretation, take a long time, and are expert-dependent (31). Furthermore, some techniques are not scaled up easily to handle a large amount of heterogeneous data, especially in real-time clinical settings (32). Current systems such as ICDs and S-ICDs are reactive in nature and are only capable of detecting arrhythmic events after they have already occurred; therefore, they are less applicable toward the mitigation of life-threatening arrhythmic incidents (33). Moreover, most machine learning models would still use handcrafted features of ECG data, and it is well understood that this type of feature may not be able to capture more complex patterns as required to accurately detect arrhythmia and possibly even more so because some arrhythmia events (such as VF) are rare (34).

To add to the burden, detection systems that operate using multi-lead ECG data challenge the effectiveness of models such as CNNs and LSTMs where such systems have to learn intricate connections between the leads (35). The models can easily overfit since the cases are often small-scale and imbalanced as in a clinical setting. The issue of unequal class distribution is particularly noticeable since the probability of life-threatening arrhythmias is significantly lower than the regular heart rate, so they are under-predicted. Such concerns are of significance in terms of developing more scalable, efficient, and accurate models that can deal with large, complex datasets and yield timely, real-time arrhythmia identification to be used in clinical practice.

## 3 Material and method

**Figure 1:**
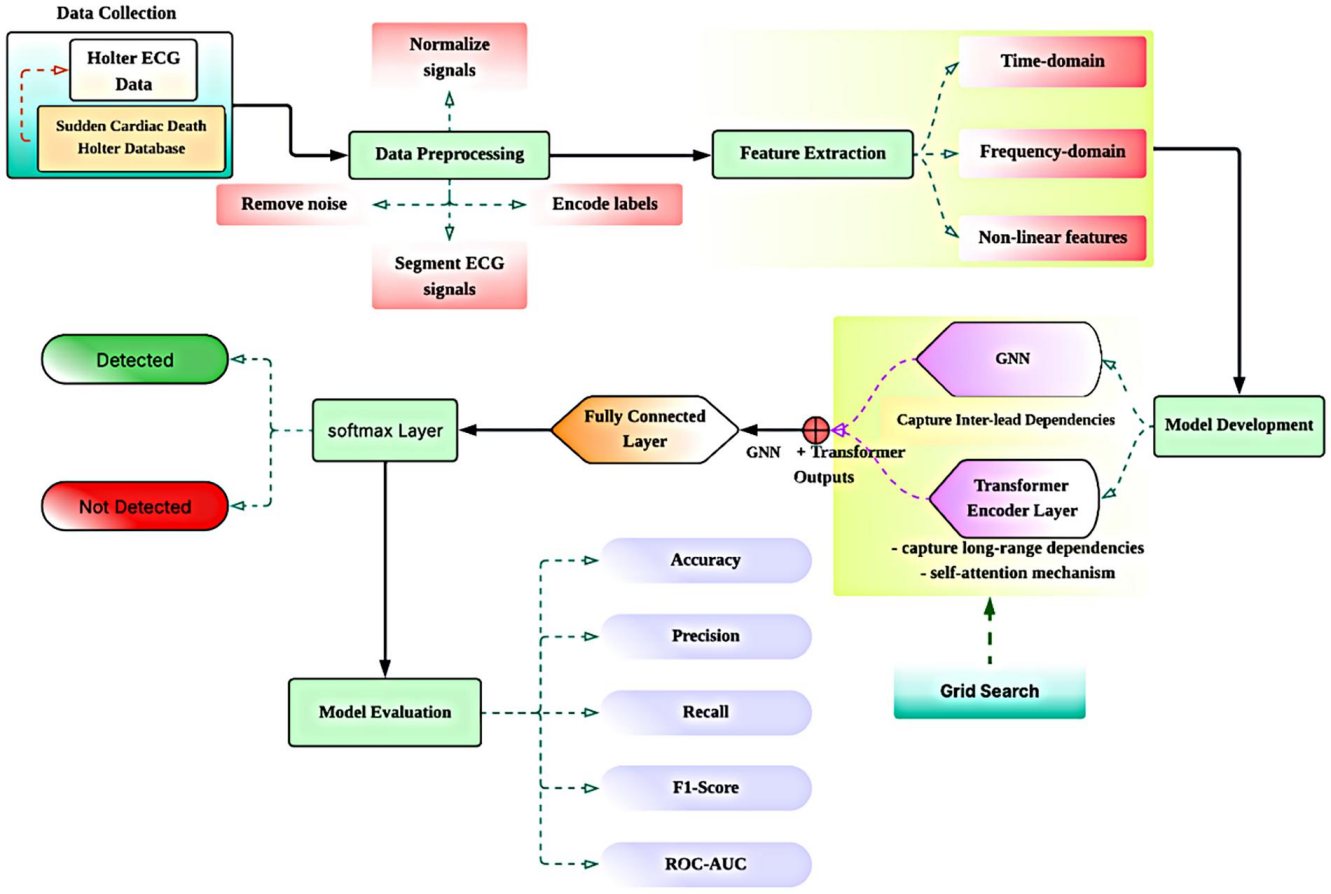
Workflow of GNN-transformer-based arrhythmia detection system.

To address the difficulties related to successfully identifying the life-threatening arrhythmias using Holter ECG data, particularly with regard to such complications as the complexity of the multi-lead nature of the data and the temporal relationships between the measurements, the example of the GNT-ArrhythmiaNet hybrid model workflow is presented in Figure 1. It starts with the collection of the data in the Holter ECG and SDDB. The data are then subjected to preprocessing involving steps such as deletion of noise, normalization of signals, encoding labels, and segmentation. This is followed by feature extraction based on time-domain, frequency-domain, and non-linear features. The processed features are processed with a hybrid model and a combination of GNNs used to capture the relationships between the leads and transformer encoder layers used to capture long-range temporal dependencies and produce self-attention. A fully connected layer and softmax layer are used to categorize the output of the model and discovered measures to evaluate the obtained outcomes validate the F1-score, ROC-AUC, recall, accuracy, and precision. The pipeline ends with grid search hyperparameter optimization, which can guarantee the maximum performance of detecting arrhythmia as exemplified in Figure 1.

### 3.1 Data collection

Data collection will concentrate on collecting Holter ECG data on a reliable and established database, namely, the SDDB, and other sources of Holter ECG data that are taken from the SDDB dataset. The SDDB is a database with ECG signal recordings of patients with a risk of developing sudden cardiac death, which will be valuable data to detect arrhythmia. The datasets are crucial because they offer real-world data on ECG signals, containing a wide range of sorts of arrhythmias, ventricular tachycardia, and fibrillation in particular, for which the model training is vital. The obtained datasets also facilitate a better representation of ECG signals and their respective arrhythmic events, which is also essential in producing a robust model that can effectively generalize well in various patient groups.

Additionally, many studies have used the data that are continuously recorded on the Holter ECG of SDDB and, therefore, are valuable to the diagnosis of subtle changes in arrhythmias occurring over the long term. These data are partitioned, over R-peak locations, or fixed time intervals to permit careful examination of brief sections of ECG. By including ECG leads in the SDDB, the model can be exposed to the dependency that exists in the spatial arrangement of the various leads, and this aspect is crucial when relating to the identification of multi-lead arrhythmias. The second reason is the relevance of SCD in this dataset to the prediction of life-threatening situations in arrhythmia, which improves the clinical use of the constructed model (dataset link: https://physionet.org/content/sddb/1.0.0/).

### 3.2 Data preprocessing

Preprocessing of the data takes a number of important steps to train the models using raw ECG signals. First, the signals are normalized so as to maintain consistency and also remove any form of scaling between various recordings. The noise removal techniques are then used to eliminate irrelevant signals, i.e., baseline wander or motion artifacts. Lastly, windows of fixed length are used to obtain segments of the ECG signals, and the labels are serialized, so the data can be ready to undergo feature extraction and entered into models.

#### 3.2.1 Remove noise

Removal of noise is a relevant step toward the enhancement of the ECG signals by eliminating the unwanted signal. The most typical methods of removing noise are bandpass filtering and high-pass filtering. A bandpass filter may be identified by Equation 1.

$$H(f)=\{\begin{array}{cc} 1\; & \;\mathrm{for}\;f_{\mathrm{low}\;}\leq f\leq f_{\mathrm{high}\;}\;\\ 0\; & \;\mathrm{otherwise}\;\; \end{array}$$ (1)

In this study, a fourth-order Butterworth bandpass filter with a lower cutoff frequency of 0.5 Hz and an upper cutoff frequency of 40 Hz was applied. The lower cutoff removes baseline wander and motion artifacts, while the upper cutoff suppresses high-frequency (HF) noise such as muscle activity and power-line interference, preserving clinically relevant ECG components.

#### 3.2.2 Normalize signals

Normalization brings down the amplitude of the ECG signal to the same scale basis so that all elements of the input do not dwarf the learning process on some characteristic features. Probably the most well-known of these strategies is the min–max normalization, that is, transposing the ECG signal x(t) to the unit interval [0,1] using Equation 2.

$$x_{\mathrm{norm}\;}(t)=\frac{x(t)-\min(x)}{\max(x)-\min(x)}$$ (2)

where min(x) and max(x) are the minimum and maximum values of the signal, respectively.

#### 3.2.3 Segment ECG signals

To make learning feasible, segmentation is performed by breaking long Holter ECG recordings into shorter sequences of fixed length. This step allows the model to focus on local temporal patterns within a specified interval and ensures a consistent input size for both feature extraction and transformer-based sequence modeling. In this study, ECG signals were segmented into fixed-length windows of Δt  = 10s. Formally, the nth ECG segment $S_{n}$ extracted from the continuous signal x(t) is formally defined in Equation 3.

$$S_{n}=\{x(t):t\in [n\cdot \Delta t,(n+1)\cdot \Delta t]\}$$ (3)

where Δt denotes the segmentation window duration, fixed to 10s in this study, and *n* is the segment index. Given the sampling frequency $f_{s}$, each segment contains $N=f_{s}\times \;\Delta t$ samples (e.g., *N*  = 3,600 samples when $f_{s}$  = 360 Hz). A sliding-window strategy with 50% overlap was employed, corresponding to a stride of 5s (N/2), to increase the number of training samples while preserving temporal continuity between adjacent segments.

#### 3.2.4 Encode labels

Label encoding is used to transform the categorical types of arrhythmias into numbers so that they can be compatible with the models. In the case of a multi-class task, the labels of the different types of arrhythmias *y* are mapped to integer *k*, where *k* is the index of a class. Television symbol coding could be expressed as Equation 4.

$$y_{\mathrm{encoded}\;}=\;\mathrm{label}\_\mathrm{map}\;(y)$$ (4)

where label_map(y) is a dictionary or mapping that assigns a unique integer to each class.

### 3.3 Feature extraction

The concept of feature extraction means calculating significant attributes that aid in the sensitivity of the model to detecting the arrhythmia in the raw ECG signals. This involves analysis of the time domain of features such as heart rate and RR interval which detects simple characteristics of the ECG waveform. Moreover, frequency-domain features, such as power spectral density (PSD), are calculated to analyze the frequency components of signal. More complex and hidden patterns in the ECG data are also extracted by means of non-linear features. Such features are essential in feeding the model with valuable and informative data to differentiate between normal and arrhythmic events.

#### 3.3.1 Time-domain features

Time domain characterizes the ECG signal in relation to the time aspect with reference to individual heartbeats. Some of the common time-domain features are the HR, RR interval, and QRS duration. As an illustration, the RR interval between R-peaks is calculated using Equation 5.

$$\mathrm{RR}\;\mathrm{Interval}\;=t_{R(i+1)}-t_{R(i)}$$ (5)

where $t_{R(i)}$ and $t_{R(i+1)}$ are the time instances of two consecutive R-peaks. These features help in understanding the regularity and rhythm of the heart.

#### 3.3.2 Frequency-domain features

Features in the frequency-domain represent the frequency content of ECG signal that are used to find periodic properties and the underlying rhythms. An example of such common frequency domain features is PSD which shows the distribution of the power of a signal at the various frequencies in terms of power. Through the fast Fourier transform (FFT) method, PSD of the ECG signal x(t) is computed using Equation (6).

$$\mathrm{PSD}(f)=|\mathrm{FFT}(x(t)){|}^{2}$$ (6)

where *f* represents frequency and the FFT transforms the time-domain ECG signal into the frequency domain, revealing periodicities and frequency bands associated with arrhythmias.

#### 3.3.3 Non-linear features

Non-linear characteristics reflect powerful activity in the ECG signal beyond a linear approach such as those used by time-domain or frequency-domain characteristics. Entropy, one of such features, is a measure of unpredictability of the signal. The commonly used approximate entropy (ApEn) is determined using Equation (7).

$$\mathrm{ApEn}(m,r)=\frac{1}{N-m}\overset{N-m}{\underset{i=1}{\sum }}\;\ln\frac{d_{i}(m,r)}{d_{i+1}(m,r)}$$ (7)

where *m* is the embedding dimension, *r* is the tolerance, and $d_{i}(m,r)$ is the distance between vectors. High entropy indicates a more complex or irregular signal, which is common in arrhythmic events.

### 3.4 Model development using the GNT-ArrhythmiaNet

The model development process then uses the strength of GNNs and transformer structures as they are able to deal with the unknown variability of a multi-lead ECG signal in recognizing arrhythmia activities. GNNs are used to learn the spatial correlations between the various ECG leads to allow the model to learn the dependencies that are vital when performing arrhythmia classification accurately. In the meantime, the transformer layers could capture long-range temporal dependencies and therefore are used to capture the sequential patterns within the ECG signals, which play a significant role in detecting the arrhythmic events. Self-attention mechanism as a part of the transformer enables the model to pay attention to only the most important parts of the ECG signal, positively influencing the precision of the detection of potentially life-threatening arrhythmias. Such a hybrid architecture is fused with the advantages of both GNN and transformer models, and the model will be able to support the complexity of ECG data as well as make predictions in a robust and real-time manner.

**Figure 2:**
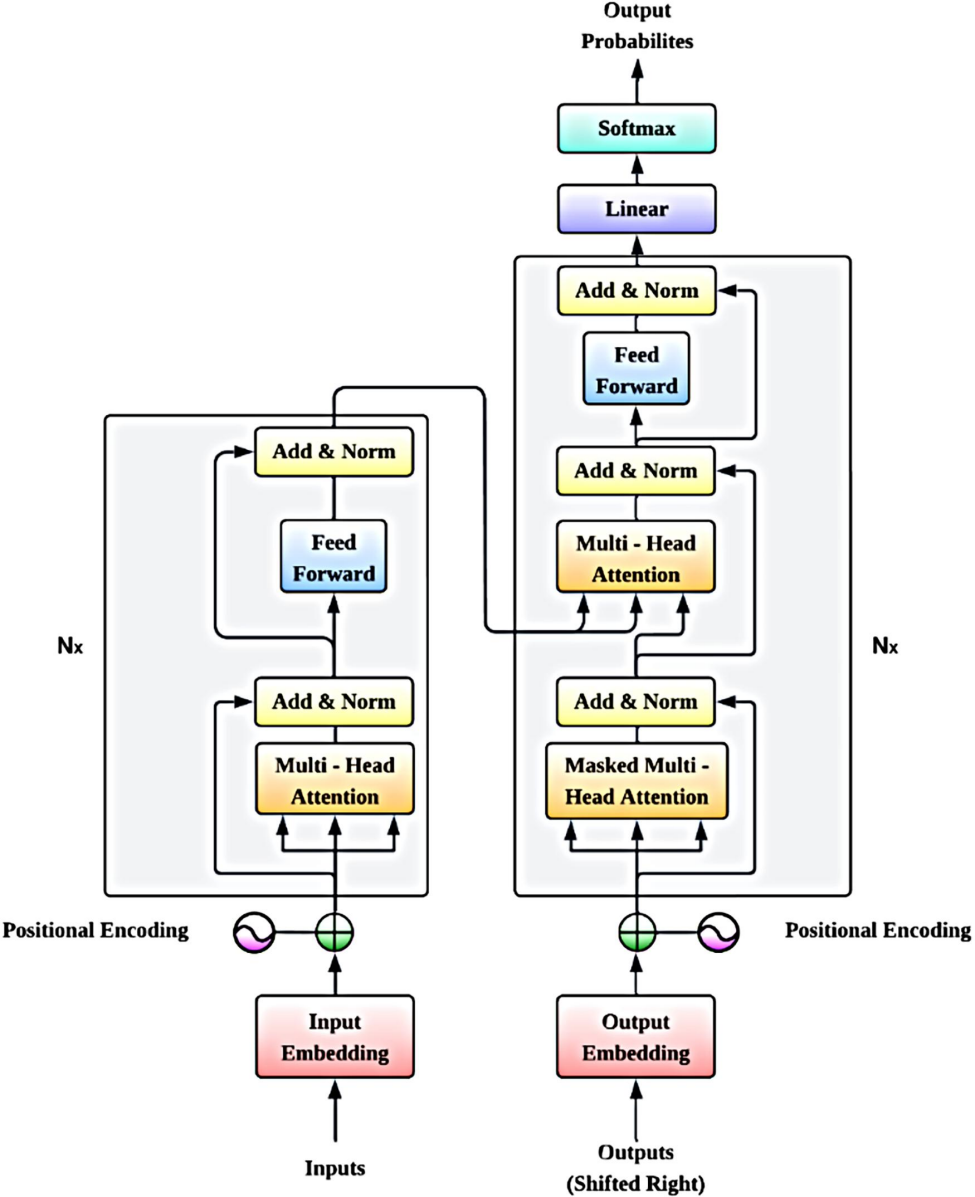
GNT-ArrhythmiaNet hybrid model architecture for arrhythmia detection.

The input ECG signals are embedded and then sent through positional encoding to preserve the temporal order of the data. The model incorporates a stack of multi-head attention layers, with add-and-norm and feed-forward blocks to appreciate spatial (lead-wise) and temporal dependencies of the ECG signals. This means that the masked multi-head attention mechanism would allow us to attend only to the information belonging to the past during autoregressive tasks. These are then fed to the output embedding which comprises a linear layer and a softmax layer that ultimately results in obtaining output probabilities used to identify when the ECG signal is detected or not detected with the presence of arrhythmias. This architecture, shown in Figure 2, integrates the advantages of GNNs and transformers by making it possible to detect arrhythmias in multi-lead electrocardiogram data effectively and correctly. This diagram describes the structure of the GNT-ArrhythmiaNet hybrid model that is used to detect arrhythmias based on a hybrid combination of GNNs and transformer networks.

### 3.5 GNT-ArrhythmiaNet hybrid model

GNT-ArrhythmiaNet is a GNN combined with transformer modules designed to enhance the detection of arrhythmia in a multi-lead ECG signal. The first step will consist of embedding the ECG signals *X* by an embedding layer and then positional encoding P to maintain the time structure. The GNN input is used as a graph G=(V,E), where *V* is the set of nodes (ECG leads) and *E* is the set of edges between the leads. The GNN trains the spatial relations among leads which are updated using the graph convolution operation shown in Equation 8.

$$h_{v}^{\left(l+1\right)}=\sigma \left(\underset{u\in N(v)}{\sum }\;\;\frac{1}{c_{vu}}W^{\left(l\right)}h_{u}^{\left(l\right)}+b^{\left(l\right)}\right)$$ (8)

where $h_{v}^{\left(l\right)}$ represents the feature vector for node *v* in the *l* -th layer, N(v) is the set of neighbors of node *v*, and $W^{\left(l\right)}$ and $b^{\left(l\right)}$ are the weight matrix and bias for the layer, respectively. Following the GNN layers, the transformer’s multi-head attention mechanism is applied to record long-term temporal dependencies, as shown in Equation 9.

$$\mathrm{Attention}(Q,K,V)=\mathrm{softmax}\left(\frac{QK^{T}}{\sqrt{d_{k}}}\right)V$$ (9)

where *D* is the dimensionality of the queries and *Q*,K, and *V* represent the queries, keys, and the values, respectively. This focus enables the model to focus on the most crucial components of the ECG signal. Once the attention mechanism is through, the model utilizes a fully connected layer to produce class probabilities. The classification is performed by the softmax function in the final classification as shown in Equation 10.

y^=softmax(Wohfinal+bo) (10)

where $h_{\mathrm{final}\;}$ is the output from the last layer and $W_{o},b_{o}$ are the output weight matrix and bias, respectively. Cross-entropy loss is used to train the model, as shown in Equation 11.

L=−∑i=1Nyilog(y^i) (11)

This hybrid model can be seen as a combination of the collective power of GNNs to consider spatial correlations between ECG leads and transformer networks to deal with their temporal correlations, and it also shows better detection rates and robustness of arrhythmia in multi-lead ECG data.

Algorithm 1 **GNT-ArrhythmiaNet**

# Input: Multi-lead ECG signal data (Holter recordings)

# Output: Predicted arrhythmia class & probabilities

def GNT_ArrhythmiaNet(input_ecg_data):

 # Step 1: Data Loading & Preprocessing

 raw_data=load_data(input_ecg_data) # Load ECG

 cleaned_data=preprocess_data(raw_data) # Filtering, normalization

 # Step 2: Feature Extraction

 features=extract_features(cleaned_data, method="time_freq_nonlinear”)

 # Shape: [num_segments, num_leads, feat_dim]

 # Step 3: Initialize Models

 gnn=initialize_GNN_model() # Spatial dependencies between leads

 transformer=initialize_transformer_model() # Temporal context learning

 # Step 4: Spatial+Temporal Modeling

 spatial_features=gnn(features) # per-segment lead relationship encoding

 temporal_features=transformer(spatial_features) # sequence modeling

 # Step 5: Classification

 logits, probs=classify(temporal_features) # Output logits+softmax probabilities

 prediction=decode_prediction(logits) # Returns label: “Normal”, “Ectopic”, etc.

 # Step 6: Decision Mapping

 if prediction == “Normal": return “Normal Sinus Rhythm”, probs

 elif prediction == “Ectopic": return “Ectopic Beat Detected”, probs

 elif prediction == “Supraventricular": return “Supraventricular Arrhythmia”, probs

 elif prediction == “Ventricular": return “Ventricular Arrhythmia”, probs

 return “Error: Unknown Prediction”, probs

def load_data(path): return data

def preprocess_data(data): return cleaned_data

def extract_features(data, method): return features

def initialize_GNN_model(): return gnn_model

def initialize_transformer_model(): return transformer_model

def classify(features): return logits, probabilities

def decode_prediction(logits): return predicted_label

End

The GNT-ArrhythmiaNet accepts the input multi-lead Holter ECG recordings and treats them with a hybrid spatial-temporal deep learning pipeline as shown in Algorithm 1. The raw and the raw ECG are pre-filtered and normalized to eliminate the noise and baseline change aspect of the signal, and then features such as time, frequency, and the aspects of the non-linear domain over segmented beats or windows are extracted. Spatial dependencies between ECG leads are modeled by a GNN, where long-range temporal patterns across the sequence are captured by a transformer encoder. The result is fused spatial-temporal representation fed to a classifier, which provides class logits and softmax probabilities and is converted to labels of clinical significance (normal sinus rhythm, ectopic beat, supraventricular arrhythmia, ventricular arrhythmia), returning the prediction and its confidence. This architecture fits in with your GNN and transformer hybrid model of robust arrhythmia detection that you proposed.

Model evaluation was performed using multiple validation folds to assess generalization performance. ECG segments were generated from continuous Holter recordings and assigned consistently within each fold to prevent overlap between training and evaluation phases. While segment-level separation was ensured, we acknowledge that strict patient-level separation remains a limitation of the current dataset and is addressed in the Discussion.

### 3.6 Hyperparameter tuning using grid search

Hyperparameter tuning using grid search is a systematic way of evaluating a list of desired hyperparameters as a way to maximize the model's performance. The search is performed by specifying a lattice of hyperparameter settings, including learning rate η, batch size *B*, and number of layers *L*. Over every combination of these hyperparameters, model training is conducted on the model, and the performance is assessed based on a validation set. It is to be minimized by a loss function, an example being the cross-entropy loss as shown in Equation 12.

L=−∑i=1Nyilog(y^i) (12)

where $y_{i}\;$ is the predicted probability and yi^ is the true label. Its hyperparameters are set to optimize this loss, and hence the best set is chosen. Performance of the final model is assessed using some measures to choose an optimum configuration. The grid search guarantees the most suitable arrangement of the model in the detection of arrhythmia using various datasets. The final set of hyperparameters selected through grid search is summarized in Table 1.

**Table 1:** Optimal hyperparameter configuration for GNT-ArrhythmiaNet.

Hyperparameter	Symbol	Final selected value
Learning rate	η	0.0001
Batch size	B	32
Optimizer	–	Adam
Number of GNN layers	LGNN	3
Number of transformer layers	LTr	4
Embedding dimension	dmodel	128
Number of attention heads	h	8
Dropout rate	–	0.3
Loss function	–	Categorical cross-entropy
Activation function	–	ReLU

**Table 2:** Performance comparison of GNN, transformer, and hybrid GNN + transformer model.

Metrics	GNN	Transformer	Proposed (GNN + transformer)
Accuracy	79.23%	63.45%	98.27%
Precision	96.43%	96.51%	98.08%
Sensitivity	79.23%	63.45%	98.27%
F-measure	86.75%	76.46%	97.76%

As shown in Table 1, the selected hyperparameter configuration provided the best validation performance and was therefore used consistently in all experiments

### 3.7 Fully connected layer

The proposed model's fully connected layer performs the final classification by using features that were extracted by the GNN and transformer layers. It has a connection between all the neurons of the preceding layers to every neuron of the layer which makes the model fuse the learned spatial and temporal features together. Mathematically, it operates a linear transformation of the input vector, *x*, which is then validated through an activation process *f* of the form indicated in Equation 13.

y=f(Wx+b) (13)

where *W* denotes the weight matrix, *b* is the bias, and *f* is typically a ReLU activation function. This layer allows the model to learn complex combinations of the features for accurate arrhythmia classification. The softmax layer then receives this layer's output for final classification into arrhythmia types.

### 3.8 Softmax layer

Finally, the model requires a softmax layer that is the output layer of the model in view of the multi-classification method in which the raw output scores of the fully connected layer are converted to a probability value. It also makes sure that the total probability of the outputs to all the classes is equal to 1, and, therefore, it can be used to assign confidence to each class such as normal, PVC, VT, or VF. The softmax operation exponentiates all logits and scales each of them to sum to one. Produced output is marked as Detected which means that a potentially dangerous arrhythmia has been detected. On the other hand, in case a normal class is the most likely, the output is Not Detected, represented by the fact that there is no critical arrhythmia. This dichotomous method of decision-making makes the output more arrayed toward the clinician with regard to only conditions that require prior notice. The sensitivity of detection is automatically taken care of by the probability density of the softmax. This step is critical in providing interpretable real-time decisions on the occurrence of arrhythmia.

## 4 Result and discussion

The Result and Discussion section shows the performance of the model based on the subsequent evaluation criteria: ROC-AUC, F1-score, recall, accuracy, and precision (36, 37). It examines the effectiveness with which the model identifies arrhythmias, especially life-threatening ones such as VT and VF. A comparison of the suggested solution to the current methods is also outlined in this section with improvements or shortcomings identified. The major results are explained with the background of clinical utility and the model validity.

### 4.1 Performance metrics

- Accuracy

The percentage of all right predictions the model makes out of all forecasts is known as accuracy. It illustrates how well the concept works overall in identifying both arrhythmic and normal ECG signals correctly. The formula for accuracy is demonstrated in Equation 14.

$$\;\mathrm{Accuracy}\;=\frac{TP+TN}{TP+TN+FP+FN}$$ (14)

where TP means true positives; TN, true negatives; FP, false positives; and FN, false negatives.

(1) Precision

Precision measures the proportion of correctly predicted positive cases out of all cases that the model labeled as positive. It indicates how reliable the model is when it raises an alert for a specific arrhythmia. The formula for precision is illustrated in Equation 15.

$$\;\mathrm{Precision}\;=\frac{TP}{TP+FP}$$ (15)

where TP means true positives and FP means false positives.

iii. Sensitivity

Sensitivity, sometimes referred to as recall, gauges how well the model can detect real positive cases, such as arrhythmic events when they actually happen. In medical applications, where omitting a real case can have dire repercussions, it is essential. The formula for sensitivity is given in Equation 16.

$$\;\mathrm{Sensitivity}\;=\frac{TP}{TP+FN}$$ (16)

where TP means true positives and FN means false negatives.

(1) F-measure

When there is an unequal distribution of classes, the F-measure, also known as the F1-score, provides a balanced metric by taking the harmonic mean of precision and sensitivity. It is particularly helpful when there are serious repercussions from both false positives and false negatives. The formula for F-measure is demonstrated in Equation 17.

$$F1\text{-}\mathrm{score}\;=\frac{2\times \;\mathrm{Precision}\;\times \;\mathrm{Sensitivity}\;}{\;\mathrm{Precision}\;+\;\mathrm{Sensitivity}\;}$$ (17)

The GNT-ArrhythmiaNet framework has also proven to have excellent performance with an accuracy of 0.9827, and this has affirmed its efficiency in the accurate classification of ECG signals. Its accuracy of 0.9808 shows that the probability of false positive inference is very minimal, and therefore most of the instances of arrhythmia that are inferred by the algorithm are confirmed. Sensitivity 0.9827, the other indicator, shows significant strength of the model in finding the actual cases of arrhythmia without overlooking the essential events. The F-measure, 0.9776, indicates an equal performance of precision and recall, and this allows it to be robust in undertaking the task of classification.

**Figure 3:**
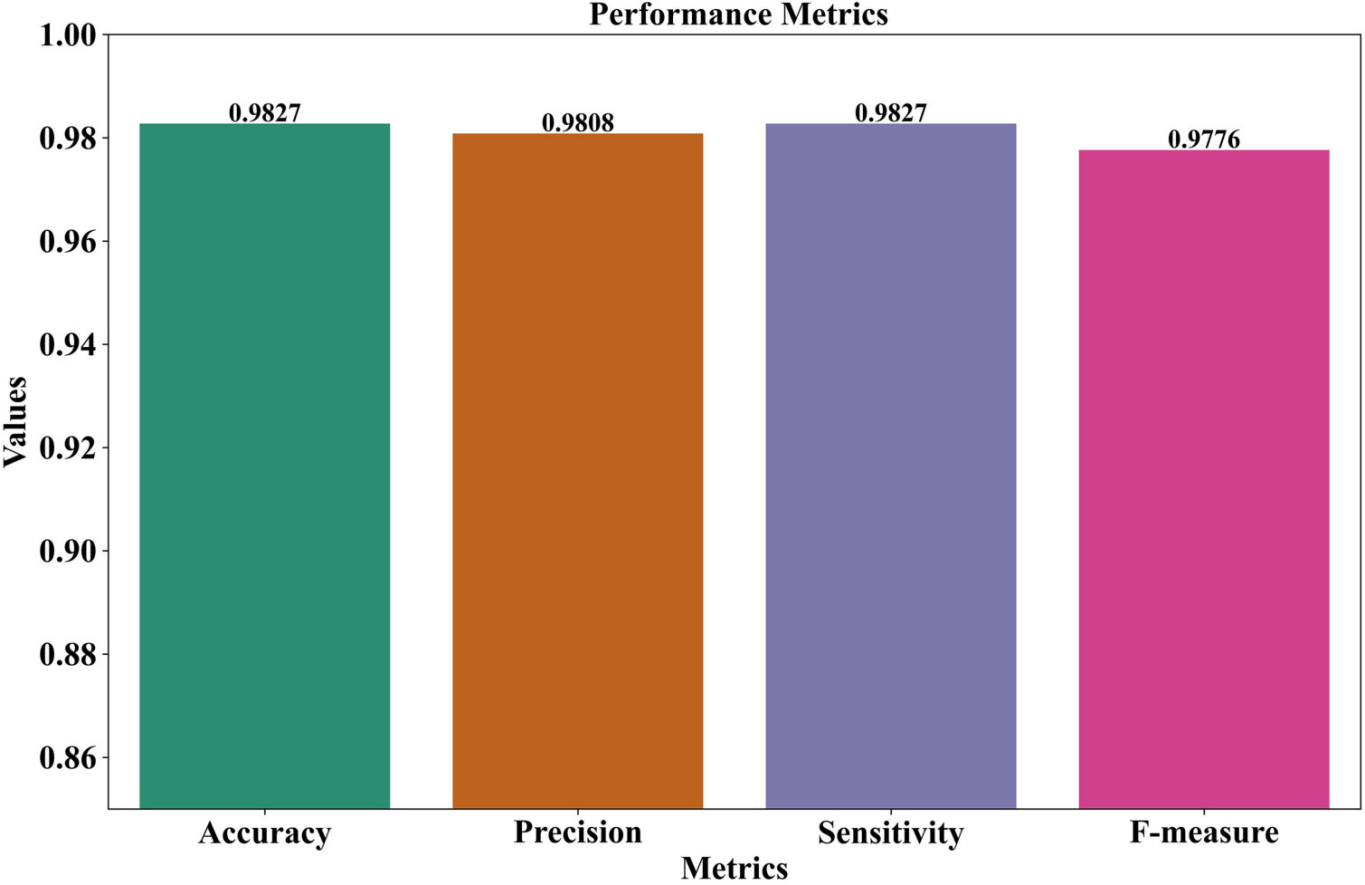
Comprehensive performance evaluation of the GNT-ArrhythmiaNet framework.

All these measures establish the viability of the framework as effective in offering reliable and consistent arrhythmia detection. These high-performance values qualify it to be used in real-life and clinical applications since accuracy and reliability are of the essence. Figure 3 further proves that the combination of GNN and transformer-based architectures in this hybrid model plays a prominent role in upgrading the classification performance concerning ECG-based arrhythmia detection.

### 4.2 Training and validation accuracy and loss performance of the proposed framework

The figures display the suggested GNT-ArrhythmiaNet model's training and validation accuracy as well as loss across the first 15 epochs. The left graph depicts a steady increase in accuracy to approximately 98.2% and 98.2% in the training and validation, respectively, signifying consistent learning with no over-fitting.

**Figure 4:**
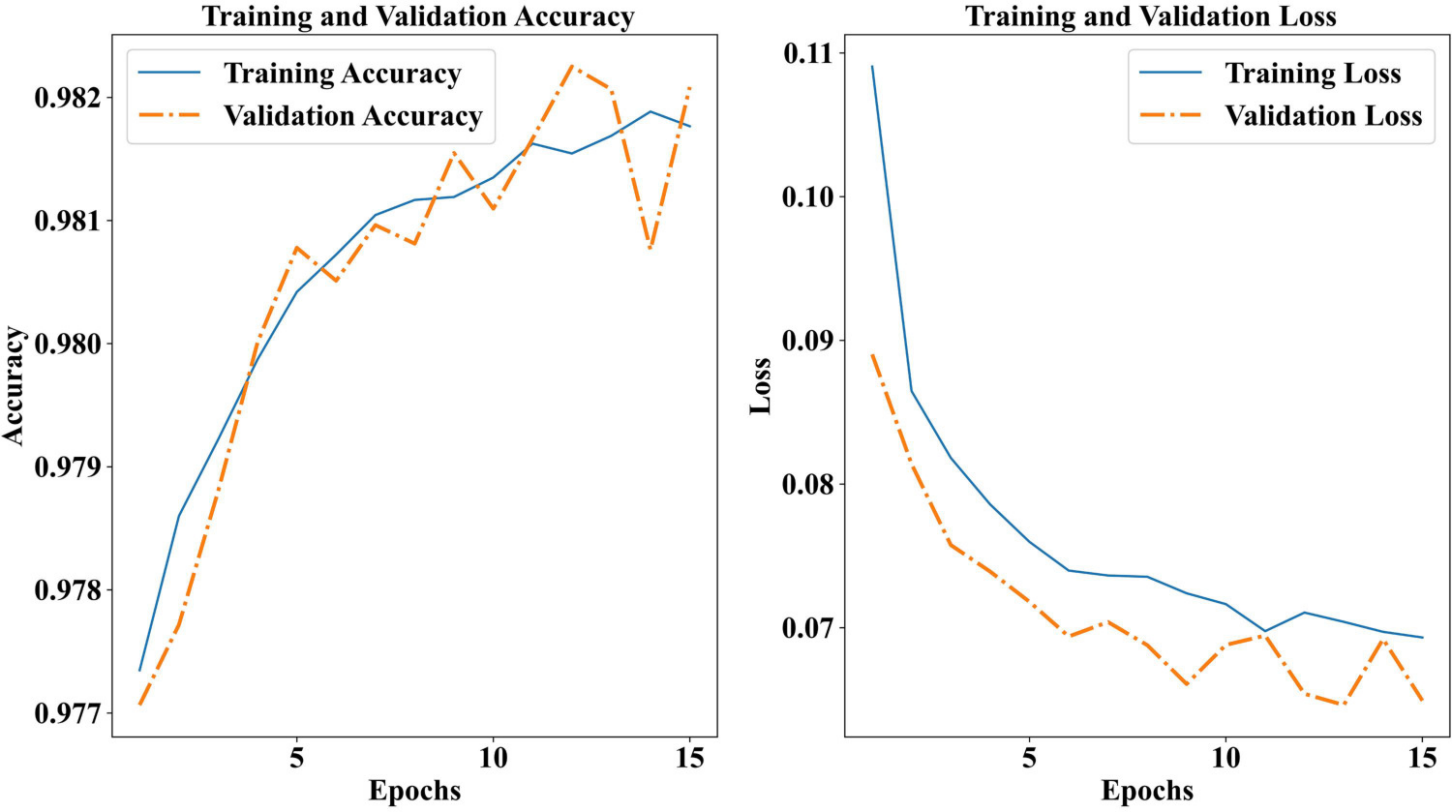
Training and validation performance of the GNT-ArrhythmiaNet model.

In the right plot, there is an evident drop in losses, approximately 0.11–0.07 in training and ever so slightly lower in validation reflecting appropriate optimization and generalization. The results show that the model works with high accuracy in both datasets with minimal errors as depicted in Figure 4.

#### 4.2.1 ROC curve analysis of GNT-ArrhythmiaNet

The proposed GNT-ArrhythmiaNet model demonstrates strong discriminative ability across multiple arrhythmia classes, with AUC scores of 0.9539 for E, 0.9565 for N, 0.9442 for S, and 0.9845 for V.

**Figure 5:**
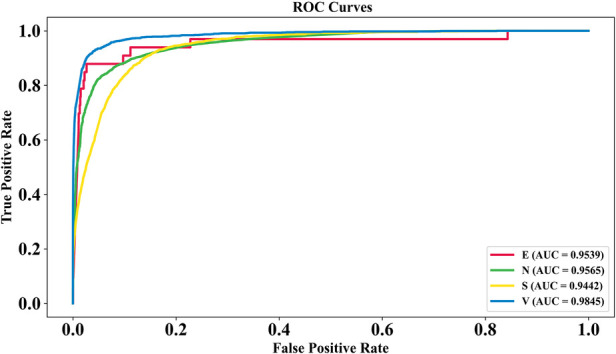
ROC curve analysis of GNT-ArrhythmiaNet for multi-class arrhythmia detection.

These high values indicate excellent sensitivity and specificity, confirming the robustness of the model in differentiating between various ECG patterns. Figure 5 validates its potential for accurate and reliable classification in clinical arrhythmia detection scenarios.

### 4.3 FNR—FPR rate for proposed work

Analyzing the GNT-ArrhythmiaNet model, one can find out that its FNR is equal to 0.1826, so the model did not miss much of the real cases of arrhythmia. This low FNR indicates its high capabilities to correctly identify the cases of the true positive, which is crucial in the medical context of diagnostics in critical cases.

**Figure 6:**
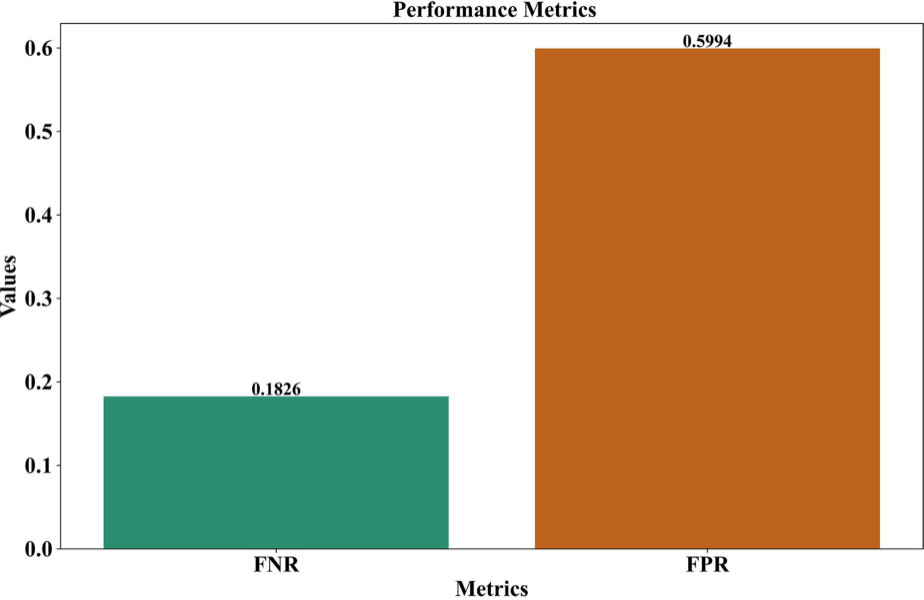
Error rate assessment of the GNT-ArrhythmiaNet framework.

On the other hand, FPR is 0.5994 which indicates that a larger amount of non-arrhythmic cases were found to be indicated as wrong arrhythmia. Although the low FNR illustrates its clinical usefulness in reducing the number of missed diagnoses, the high FPR implies that the poor performance in real-time applications will tend to over-detect and receive unnecessary alerts. This kind of false positive can affect the usability of the systems, especially in continuous or wearable monitoring. As shown in Figure 6, focusing on the accuracy in the detection feature of the model, it is pertinent to continue optimizing to maintain sensitivity and specificity. In general, the decrease of FPR and preservation of sensitivity will achieve a more feasible and reliable system with the final deployment in real-world healthcare conditions.

### 4.4 Confusion matrix analysis of GNT-ArrhythmiaNet

These results of classification indicate that the model was not mistaken when it came to labeling ectopic beats as “E” since it never mixed them with other classes, and that 32 were falsely classified as normal as “N” and 1 falsely as ventricular as “V.” “N” had the most correct classification number at 145,972 of which 39 had been incorrectly labeled as supraventricular beats N and 81 as N. Of ‘S, 1,706 were erroneously classified as ‘N, 285 as the same class as itself, S, because confused with adjacent morphology and 14 as ‘V. There were 706 misclassified as N in the case of V, one as S and 605 as correctly classified.

**Figure 7:**
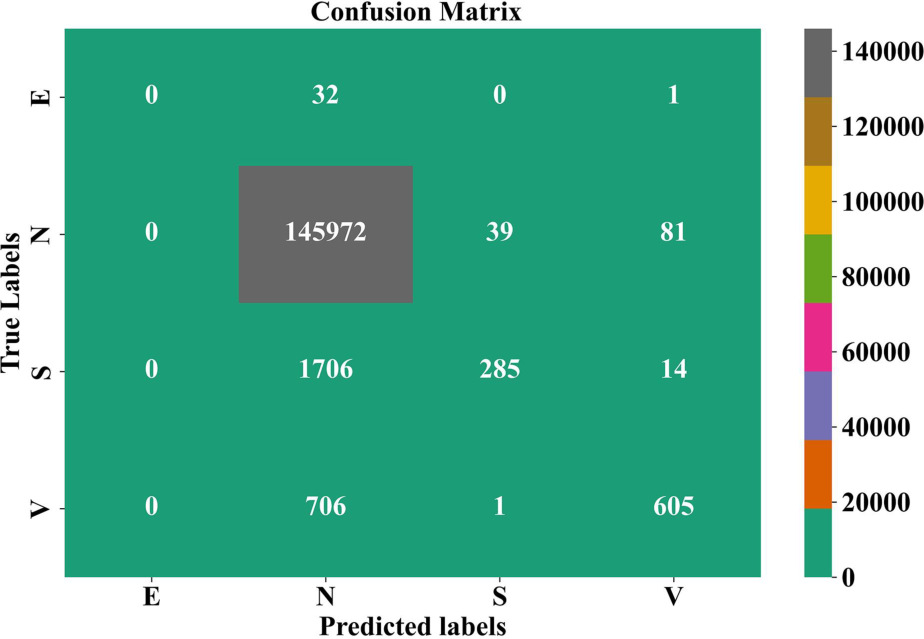
Confusion matrix analysis of the GNT-ArrhythmiaNet framework.

These results indicate that while normal beats dominate the dataset with high classification accuracy, the model faces challenges in correctly identifying supraventricular and ventricular beats due to morphological similarities in ECG patterns. Figure 7 highlights the need for enhanced feature extraction techniques to better distinguish minority arrhythmic classes.

It is important to note that the overall classification accuracy of 98.27% reflects aggregate performance across all classes and is influenced by the dominance of normal and majority rhythm classes. Life-threatening arrhythmias such as VF and VT are significantly underrepresented in the dataset, which contributes to reduced classification performance for minority classes, including supraventricular beats. Although the proposed GNN + transformer architecture improves feature discrimination through attention-based temporal modeling, no explicit data-level balancing or synthetic augmentation was applied in the current implementation. Therefore, class-wise metrics and confusion matrix analysis provide a more accurate representation of model behavior for rare arrhythmic events.

### 4.5 Feature evaluation in proposed GNT-ArrhythmiaNet

The top-left analysis of the ECG segment shows precise R-peak detection on both raw and cleaned ECG signals, with noise effectively removed in the cleaned version, enhancing the clarity of waveform components. Stable amplitude is observed throughout most of the segment, but distinct fluctuations appear approximately the 60 s mark, aligning with notable cardiac events. Signal quality remains high across the duration except during short noisy intervals, ensuring dependable data for feature extraction. The bottom-left heart rate plot reveals beat-to-beat variations between ∼60 and ∼95 bpm, with a mean rate near 80 bpm, while sharp spikes correspond to potential arrhythmic triggers. This consistent rate monitoring offers valuable insight into cardiac rhythm dynamics. The right-hand morphological analysis displays over 300 aligned heartbeats, with P, Q, S, and T waves distinctly color-coded, and the average beat shape emphasizing a pronounced R-wave peak and clearly identifiable P- and T-wave morphology.

**Figure 8:**
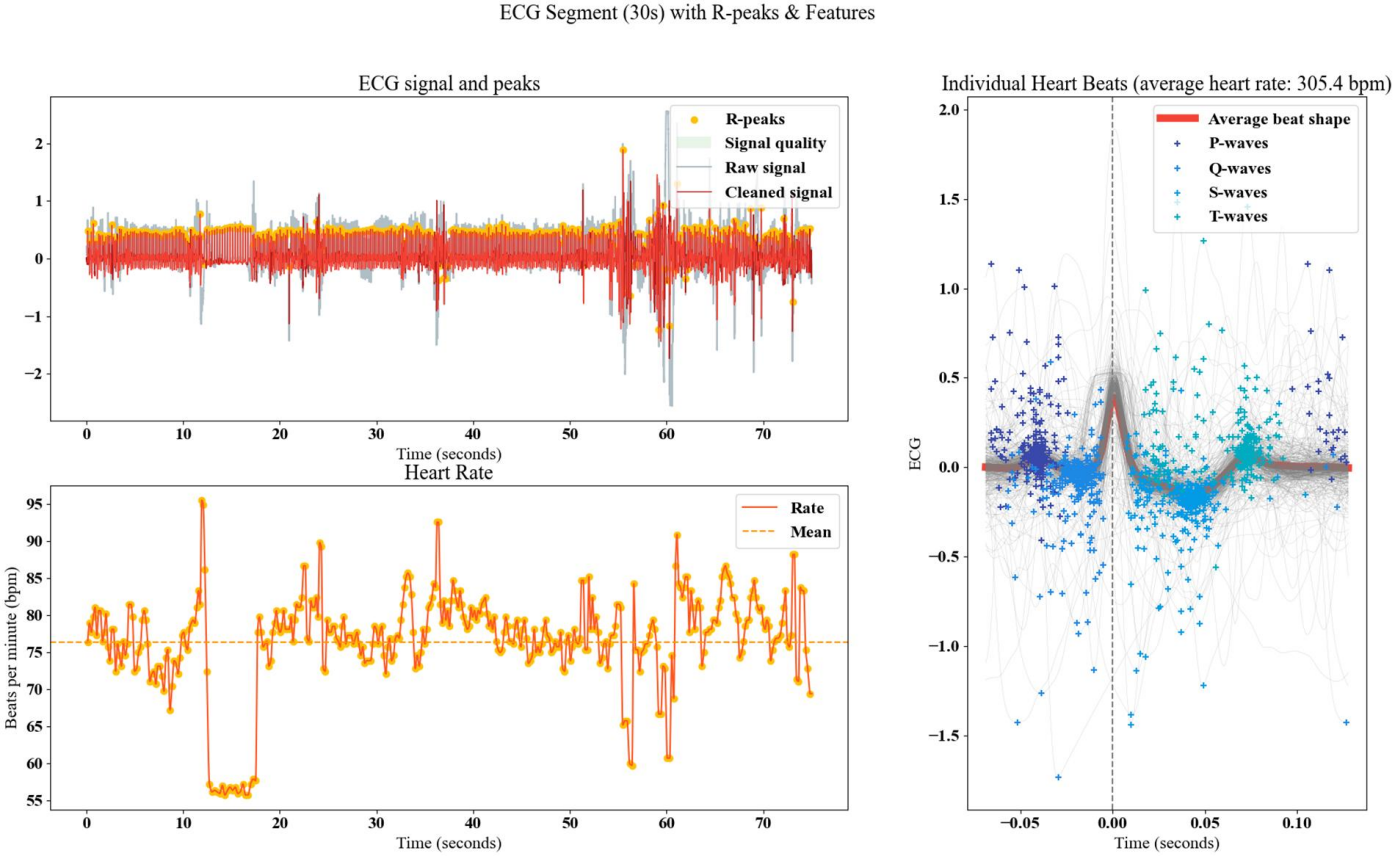
Comprehensive ECG signal, rate, and morphological feature evaluation in GNT-ArrhythmiaNet.

The calculated average heart rate of 305.4 bpm highlights severe tachyarrhythmia, reflected in the densely packed QRS complexes and minimal shape variability, forming a critical input for GNT-ArrhythmiaNet's precise arrhythmia classification as illustrated in Figure 8.

### 4.6 Poincaré-based RR interval variability in proposed work

The distribution of RR intervals reveals a dense clustering between 750 and 850 ms for both the current (*n*) and successive (*n* + 1) beats, indicating a generally stable heart rhythm with moderate variability. Outliers are visible in the higher range, particularly above 1,000 ms, suggesting occasional prolonged intervals that could signify arrhythmic pauses or compensatory beats. The compact central cluster implies a low standard deviation of normal-to-normal intervals (SDNN), aligning with a relatively steady cardiac rhythm. Sparse data points at the lower end (∼650 ms) correspond to episodes of faster heart rates, possibly due to transient tachycardia.

**Figure 9:**
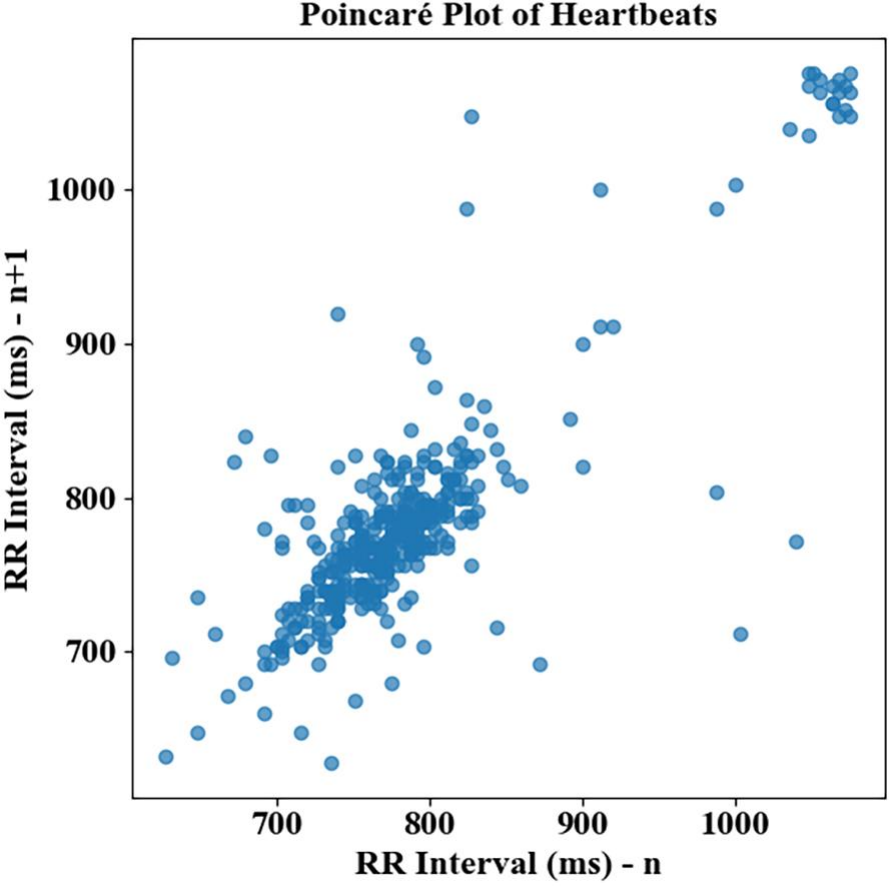
Poincaré-based RR interval variability assessment in GNT-ArrhythmiaNet.

Figure 9 suggests controlled variability suitable for healthy function, but the presence of extreme values highlights the importance of continuous monitoring for sudden changes. This variability profile forms a critical time-domain feature input for the GNT-ArrhythmiaNet classification process.

### 4.7 Heart rate variability profiling in GNT-ArrhythmiaNet

The distribution of RR intervals reveals a dominant clustering approximately 780–820 ms, forming a near-normal curve with minimal skewness, while outliers at both extremes suggest occasional tachycardia and bradycardia events. The PSD analysis highlights the predominance of very low-frequency (VLF) and low-frequency (LF) components, with LF power contributing most to autonomic balance and a smaller HF component reflecting parasympathetic activity.

**Figure 10:**
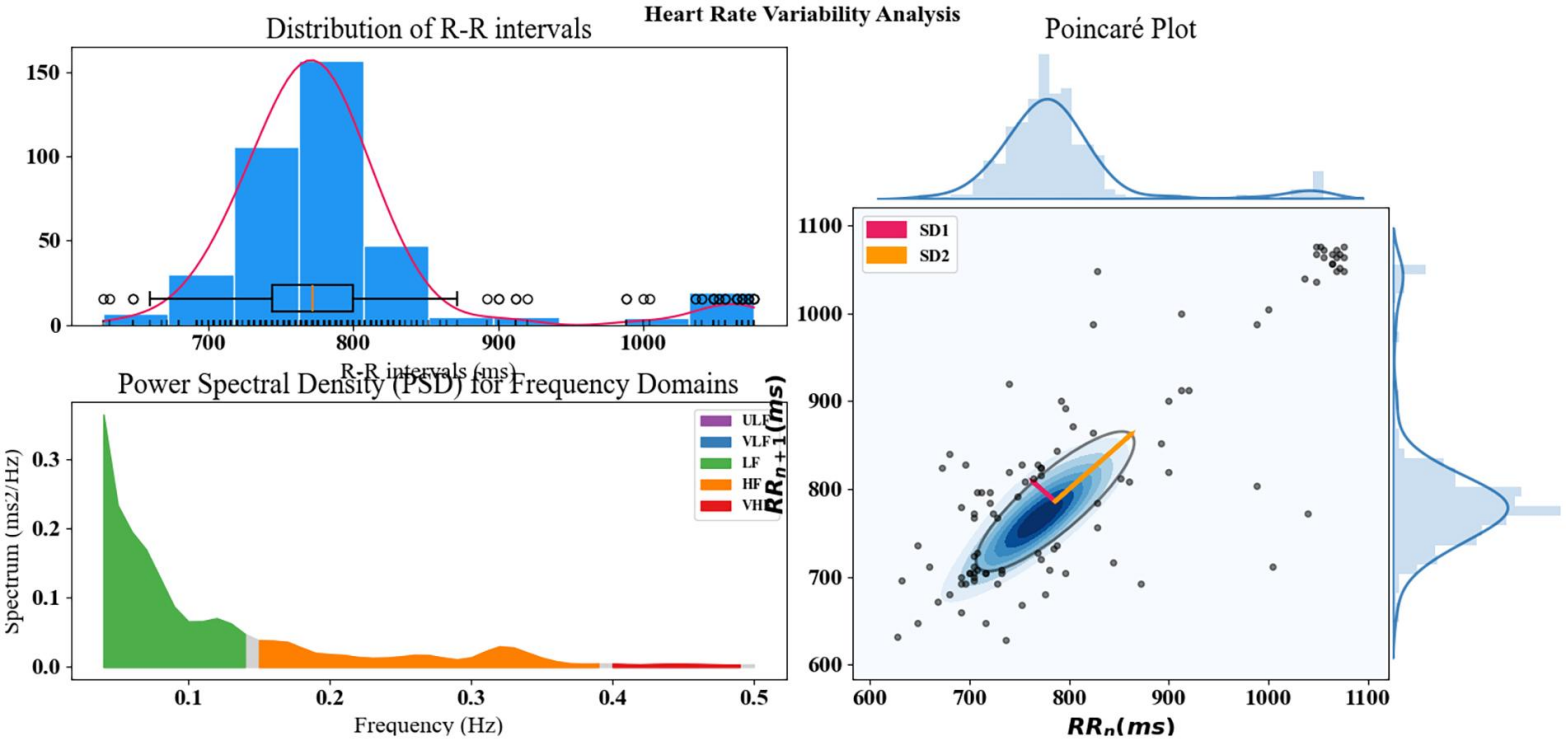
Comprehensive heart rate variability profiling in GNT-ArrhythmiaNet.

The Poincaré plot shows a dense elliptical cluster along the line of identity with SD1 and SD2 values indicating low short-term variability, and the variability pattern is generally stable and well-regulated, with moderate long-term variability consistent with a stable rhythm but with notable outliers extending beyond 1,000 ms. Together, Figure 10 confirms that while the heart rate variability pattern is generally stable and well-regulated, sporadic deviations exist, emphasizing the value of integrating such variability metrics into the GNT-ArrhythmiaNet framework for improved arrhythmia detection and classification.

### 4.8 Ablation analysis

Ablation analysis is a technique utilized to assess the effects of different components or features of a model by systematically removing or modifying them. It helps understand how each part of the model contributes to its overall performance. By comparing the results before and after removing or altering components, ablation analysis identifies key elements that improve or hinder the model's effectiveness. This process is crucial for optimizing the model and ensuring that all components are necessary and contribute positively to its success.

The model's performance dramatically increases when both the GNN and transformer components are combined. The GNN-only model achieves an accuracy of 79.23%, a precision of 96.43%, and a sensitivity of 79.23%, with an F-measure of 86.75%. The transformer-only model performs slightly worse, with an accuracy of 63.45%, precision of 96.51%, and sensitivity of 63.45%, showing its limitations in detecting arrhythmias without the GNN's spatial feature learning. However, the combined GNN and transformer model excels with an accuracy of 98.27%, precision of 98.08%, sensitivity of 98.27%, and an F-measure of 97.76%, demonstrating the enhanced capability of the hybrid approach to effectively identify and classify arrhythmias. Table 2 clearly highlights the importance of integrating both models for improved performance.

**Table 3:** Effect of hyperparameter optimization on classification metrics.

Hyperparameter Tuning	Accuracy (%)	Precision (%)	Recall (%)	F1-score (%)
With hyperparameter tuning	98.27%	98.08%	98.27%	97.76%
Without hyperparameter tuning	94.12%	95.02%	93.22%	94.50%

The model demonstrates a notable improvement with hyperparameter tuning, achieving a 98.27% accuracy, precision of 98.08%, and recall of 98.27%, with an F1-score of 97.76%. In contrast, when hyperparameter tuning is not applied, the performance drops significantly, with an accuracy of 94.12%, precision of 95.02%, and recall of 93.22%, resulting in an F1-score of 94.50%. This indicates that fine-tuning the hyperparameters increases the capacity of the model to correctly classify arrhythmias and minimizes errors, leading to better overall performance. Table 3 shows the importance of hyperparameter optimization in improving model effectiveness, particularly for tasks involving complex ECG signal classifications.

**Table 4:** Influence of attention mechanism on model performance.

Attention Mechanism	Accuracy (%)	Precision (%)	Recall (%)	F1-score (%)	Specificity (%)
With attention mechanism	98.27%	98.08%	98.27%	97.76%	0.98%
Without attention mechanism	95.76%	94.62%	95.00%	94.81%	0.90%

The model with the attention mechanism performs significantly better, achieving a 98.27% accuracy, a precision of 98.08%, a recall of 98.27%, and an F1-score of 97.76%, with a specificity of 98%. In contrast, without the attention mechanism, the model's performance declines, with accuracy dropping to 95.76%, precision to 94.62%, and recall to 95.00%, while the F1-score decreases to 94.81% and specificity reduces to 90%. The results clearly demonstrate the critical role of the attention system in improving the model's capacity to focus on important features, leading to better classification performance. The higher specificity with attention further highlights the improved capability of the model to correctly classify negative instances, minimizing false positives. Table 4 indicates that incorporating the attention mechanism significantly improves both the sensitivity and specificity of the model, particularly in detecting arrhythmias.

**Table 5:** Impact of feature extraction methods on classification performance.

Feature Extraction Method	Accuracy (%)	Precision (%)	Recall (%)	F1-score (%)
Time + frequency + non-linear features	98.27%	98.08%	98.27%	97.76%
Time + frequency features	95.68%	94.51%	94.78%	94.64%
Non-linear features only	92.12%	91.03%	91.32%	91.17%

The model that incorporates time, frequency, and non-linear features performs the best, with a 98.27% accuracy, 98.08% precision, 98.27% recall, and an F1-score of 97.76%. Using only time and frequency features results in a decrease in performance, with accuracy dropping to 95.68%, precision to 94.51%, recall to 94.78%, and F1-score to 94.64%. The model using non-linear features only shows the lowest performance, with accuracy at 92.12%, precision at 91.03%, recall at 91.32%, and an F1-score of 91.17%. This designates that combining all three feature types greatly enhances the model's capacity to precisely identify arrhythmias. Table 5 demonstrates the importance of incorporating non-linear features along with traditional time and frequency features for optimal classification. Without the inclusion of non-linear features, the model's capacity to differentiate between various arrhythmia kinds is substantially compromised.

### 4.9 Comparison analysis

Comparison analysis involves evaluating the suggested model's performance in comparison with current techniques to highlight improvements and advantages. It typically compares metrics such as accuracy, precision, sensitivity, and other relevant performance measures. The analysis helps in understanding how the new model performs in relation to state-of-the-art approaches, providing insights into its effectiveness and potential for real-world applications. This comparison is essential for validating the model's superiority or highlighting areas that require further refinement.

According to the results of the comparison analysis, the GNT-ArrhythmiaNet model (GNN + transformer) performed better than the previous models with all major indicators. The model recorded an accuracy of 98.27% which is greater than that of Hassan et al. (98.00%) and Barbosa et al. (96.30%). The accuracy of the proposed model is 98.08%, better than the one offered by Hassan et al. (90.96%) and Barbosa et al. (94.07%). Likewise, a high sensitivity of 98.27 signifies a great power to identify arrhythmias, which is higher than earlier models (91.00% and 98.45, respectively). The trade-off between precision and recall is highly stable, as the F-measure descriptors 97.76% showed, moving ahead of the other methods (87.79% and 96.17%, respectively). The findings illustrated in Table 6 prove that the suggested model can offer better results toward the diagnosis of arrhythmia than the other available options.

**Table 6:** Performance comparison of GNT-ArrhythmiaNet with state-of-the-art models.

Authors	Accuracy	Precision	Sensitivity	F-measure
Hassan et al. (38) CNN-Bi-LSTM	98.00%	90.96%	91.00%	87.79%
Barbosa et al. (39) Bi-LSTM	96.30%	94.07%	98.45%	96.17%
Proposed (GNN + transformer)	98.27%	98.08%	98.27%	97.76%

### 4.10 Statistical significance analysis

To verify whether the observed performance improvements of the proposed GNN + transformer model are statistically significant rather than merely numerical, inferential statistical tests were conducted. Accuracy scores obtained across multiple validation folds were used for comparison. Paired *t*-tests were performed between the proposed GNN + transformer model and each baseline method (CNN-Bi-LSTM and Bi-LSTM). The null hypothesis assumed no statistically significant difference in performance between the compared models. The results indicate that the proposed model significantly outperforms both CNN-Bi-LSTM and Bi-LSTM baselines, with *p*-values of <0.05 in all cases. This confirms that the observed accuracy improvement (98.27%) represents a statistically significant gain rather than random variation.

### 4.11 Discussion

The new model, GNT-ArrhythmiaNet, which has integrated GNNs and transformers, achieves excellent results in detecting and classifying Holter ECGs in the task of discerning arrhythmias. Using either spatial (through GNNs) or temporal (through transformers) dependencies, the model successfully learns the complex patterns that characterize ECG data. The overall score of the extensive evaluation demonstrates that the model built obtained high accuracy, 98.27%, where the precision and recall scores are equal to 98.08% and 98.27%, respectively. These outcomes promise the strong predictive performance of the model with few false positives to identify arrhythmias. Moreover, an F1-score of 97.76% demonstrates a balanced recall and precision perfectly applicable to the model which leaves no doubt that it will be very successful in both identifications of true arrhythmias and the minimization of misrepresentations. These statistics evidence the effectiveness of wrapping GNN and transformer models, and it is particularly helpful when handling the complexity of ECG data, as demonstrated in the research conducted in the past.

Compared with standard machine learning and deep learning approaches, the GNT-ArrhythmiaNet model exhibits superior performance relative to baseline methods, including support vector machine, CNN-Bi-LSTM, and Bi-LSTM models. The incorporation of attention mechanisms enhances the model's ability to focus on clinically relevant ECG characteristics associated with arrhythmic events, thereby improving sensitivity (98.27%) and specificity (98%). These findings highlight the importance of attention-based modeling in suppressing irrelevant noise and emphasizing informative ECG segments. Furthermore, the demonstrated generalizability of the proposed model across multiple ECG datasets, including the MIT-BIH and SDDB databases, indicates its potential applicability in real-world clinical settings for continuous arrhythmia monitoring. The model's robustness to noisy and variable ECG signals suggests its suitability for deployment in wearable health monitoring systems capable of reliable, real-time arrhythmia detection in high-risk patients. Although explicit attention-weight visualizations are not presented, interpretability is supported through ECG morphological analysis and temporal feature evaluation (Figures 8–10), which demonstrate that the attention mechanism prioritizes clinically relevant waveform components such as R-peaks, RR-interval variability, and rhythm irregularities. These observations are aligned with recent successful applications of artificial intelligence in electrophysiology, where AI-based decision support systems have demonstrated clinical value in continuous cardiac monitoring, early arrhythmia detection, and risk stratification (40).

Moreover, hyperparameter optimization was essential in improving the performance of the model. Model accuracy was very impressive with a hyperparameter fine-tuned score of 98.27%, which is a very large jump in accuracy compared with the baseline of 94.12%. This type of tuning process assisted the model in converging and accurately at an accelerated rate by varying learning rates, sizes of the batches, and configuration of the layers. The ablation study results also demonstrate the significance of every part comprising the hybrid model. The combination of GNN and transformer layers yields an impressive increase in accuracy given that precision (98.08%) and recall (98.27%) are high, which further implies that a multi-aspect perspective is essential in accurate arrhythmia detection. Nevertheless, with such promising results, there are areas where the model can improve especially in the reduction of false positive rates and class imbalance issue on smaller categories of arrhythmia. Additional methods of data augmentation and class rebalancing might also be considered to further optimize the model when applied in problematic clinical situations. In general, it is possible to note that the presented framework should be regarded as a significant addition to the field of arrhythmia detection because it integrates simple deep learning methods that could be practically employed in health monitoring devices.

From a clinical perspective, the observed false positive rate (FPR = 59.94%) indicates a tendency toward over-detection, which may result in unnecessary alerts in continuous or wearable monitoring environments. While such false alarms can increase clinician workload and patient anxiety, the model was deliberately optimized to achieve a low false negative rate (FNR = 18.26%), as missing life-threatening arrhythmias such as ventricular tachycardia or fibrillation carries significantly higher clinical risk. To mitigate the impact of a high FPR within the current framework, several immediate methodological strategies can be applied. First, probability threshold optimization can be used to adjust the decision boundary of the softmax output, allowing a controlled trade-off between sensitivity and specificity depending on clinical deployment requirements. Second, class-weighted loss calibration can be applied to penalize false positives more strongly for non-critical classes, reducing unnecessary alerts without compromising detection of high-risk arrhythmias. Third, temporal alert aggregation can be employed, where an alert is triggered only if consecutive ECG segments are classified as abnormal, effectively suppressing isolated false positives caused by transient noise or morphological ambiguity. These strategies can be implemented without altering the core GNN + transformer architecture and provide practical mechanisms to improve system usability in real-time clinical and wearable monitoring scenarios.

Class imbalance remains a fundamental challenge in ECG-based arrhythmia detection, particularly for rare but clinically critical events such as VF and VT. While the proposed model demonstrates strong overall accuracy, the confusion matrix reveals residual difficulty in distinguishing minority classes, especially supraventricular arrhythmias, due to limited sample availability and morphological similarity. To address this limitation within the current framework, several immediate mitigation strategies are applicable. First, class-weighted loss rebalancing can be used to penalize misclassification of minority arrhythmias more strongly during training. Second, class-specific probability thresholds can be adjusted to improve sensitivity for underrepresented classes without affecting majority class stability. Third, emphasis on class-wise performance metrics rather than aggregate accuracy provides a more clinically meaningful evaluation of rare arrhythmia detection.

These measures do not require architectural redesign and can be directly integrated into the existing training and deployment pipeline.

## 5 Conclusion

The proposed GNT-ArrhythmiaNet architecture was designed to detect and classify potentially life-threatening arrhythmias from Holter ECG recordings by leveraging a hybrid combination of GNNs and transformer architectures to model spatial and temporal dependencies in the data. The model demonstrated strong performance, achieving 98.27% accuracy, 98.08% precision, 98.27% recall, and an F1-score of 97.76%, with 98% specificity and 98.27% sensitivity, highlighting its reliability and effectiveness for real-time arrhythmia detection. Compared with conventional machine learning and deep learning baselines, including CNN-Bi-LSTM and Bi-LSTM models, the proposed approach consistently showed superior accuracy and generalization across multiple datasets, notably the MIT-BIH and SDDB databases. The incorporation of the attention mechanism further enhanced the model's ability to focus on clinically relevant ECG features, improving the AUC while reducing the influence of noise. In addition, hyperparameter optimization played a significant role in improving performance, increasing accuracy from 94.12% to 98.27%, and demonstrating the effectiveness of task-specific tuning. The resulting high AUC and precision–recall values indicate that the proposed model is well-suited for deployment in wearable healthcare systems, enabling continuous monitoring of high-risk patients.

Despite these promising results, certain limitations remain. Although GNT-ArrhythmiaNet achieves high sensitivity and a low false negative rate, the elevated false positive rate reflects an inherent trade-off between early detection and alert reliability in continuous cardiac monitoring. In high-risk screening scenarios, prioritizing sensitivity is clinically justified; however, for real-world deployment, reducing false alarms is essential. The proposed mitigation strategies including decision threshold tuning, class-aware calibration, and temporal consistency filtering offer immediate and practical solutions to address this issue and enhance system usability in wearable and real-time settings. Furthermore, the model's performance is influenced by class imbalance inherent in real-world Holter ECG datasets. Rare arrhythmias such as VF and VT continue to present classification challenges, underscoring the importance of class-aware evaluation and calibration. The outlined mitigation strategies provide immediate pathways to improve minority-class reliability and enhance the clinical robustness of the proposed framework in future deployments.

## Data Availability

The original contributions presented in the study are included in the article/Supplementary Material; further inquiries can be directed to the corresponding authors.
